# A female survivor of childhood medulloblastoma presenting with growth-hormone-induced edema and inflammatory lesions: a case report

**DOI:** 10.1186/1752-1947-3-17

**Published:** 2009-01-16

**Authors:** Veronica Biassoni, Federica Pallotti, Filippo Spreafico, Ettore Seregni, Lorenza Gandola, Antonella Martinetti, Emilio Bombardieri, Maura Massimino

**Affiliations:** 1 Department of Pediatric Oncology, Fondazione IRCCS Istituto Nazionale per lo Studio e la Cura dei Tumori, Via Venezian 1, 20133 Milan, Italy; 2 Department of Nuclear Medicine, Fondazione IRCCS Istituto Nazionale per lo Studio e la Cura dei Tumori, Via Venezian 1, 20133 Milan, Italy; 3 Department of Radiotherapy, Fondazione IRCCS Istituto Nazionale per lo Studio e la Cura dei Tumori, Via Venezian 1, 20133 Milan, Italy

## Abstract

**Introduction:**

The improved survival of children with brain tumors has increased concerns about treatment-related sequelae. Growth hormone deficiency is frequently observed after craniospinal irradiation for medulloblastoma. It has been widely reported that growth hormone replacement therapy does not increase the risk of second tumors, but there are reports in the literature of growth hormone, and its downstream mediator insulin-like Growth Factor 1, having an important proinflammatory action. There are few reports, however, on the "in-vivo" induction of edema and symptomatic inflammatory lesions during replacement therapy.

**Case presentation:**

We report the case of a 7-year-old girl treated for metastatic medulloblastoma who developed growth hormone deficiency 2 years after oncological treatment.

Three months after replacement therapy, magnetic resonance imaging showed exacerbation of her brain edema, which was already present after oncological treatment. We consequently suspended the growth hormone until a new magnetic resonance image obtained 3 months later documented a reduction of the inflammatory areas. We then re-introduced somatotropin at lower doses with no further increase in brain edema in subsequent radiological controls.

**Conclusion:**

This case and its iconography suggest a strong association between growth hormone administration and the exacerbation of inflammatory reactions within the tumor bed. Replacement therapy should be carefully monitored in this particular subset of patients.

## Introduction

Survival rates for patients with malignant pediatric brain tumors have remarkably improved in recent years; the 5-year relative survival probability for all brain malignancies combined being approximately 69%, depending on the pathophysiologic and morphologic characteristics for children diagnosed in 1992 or later [1].

Due to this improvement, more attention is being focused on the possible sequelae of oncological treatments (surgery, chemotherapy and radiotherapy), among which endocrinopathies are most frequently observed in patients treated for central nervous system (CNS) tumors with growth-hormone deficit (GHD) which is often observed after irradiation. In particular, craniospinal irradiation for medulloblastoma commonly leads to GHD (67% at 10 years after cranial irradiation greater than 24 Gy, 80% when greater than 30 Gy) and primary or mixed hypothyroidism. In fact, irradiation of the hypothalamic-pituitary axis causes a characteristic pattern of hormone loss: growth hormone (GH) is usually the first to be affected, followed by the gonadotrophins, ACTH and TSH.

In the past, there was much concern about whether GH therapy might increase cancer risk, but it has now been established that GH treatment does not increase the rate of tumor recurrence in patients previously treated for primary CNS lesions [2,3], and recent guidelines recommend starting GH therapy a year after the end of the oncological treatment if there is no evidence for further tumor growth.

In addition to the oncological safety of GH, there are reports that GH and its downstream mediator, insulin-like Growth Factor 1 (IGF-1), have an important proinflammatory action [4-8]. A recent study by Pagani et al. emphasizes that the production of proinflammatory cytokines by the peripheral blood mononuclear cells is lower in GH-deficient children than in healthy, age-matched individuals, and that cytokine production is significantly increased after 3 months of GH therapy [4].

Another study on the relationship between GH and brain edema in rat brain freeze-injury models [5] revealed that GH crosses the blood-brain barrier, finding a considerable amount of GH receptors whose density is higher in the choroid plexus, hypothalamus, hippocampus, pituitary region, and spinal cord [6]. Moreover, GH administration affects the concentrations of several neurotransmitters in the cerebrospinal fluid and is probably related to post-injury edema [6]. Further interactions between GH and/or IGF-1 with the immune-inflammatory system have been documented in the literature. For example, both GH and IGF-1 increase the bactericidal capacity of human polymorphonuclear neutrophils via intracellular reactive oxygen intermediaries, as well as increasing their complement receptor expression [7]. Moreover, GH has a proinflammatory activity also modulating several vascular growth factors; IGF-1 correlates with angiogenin levels in particular, stimulating endothelial cell proliferation as well as collagen synthesis, fibroblasts, and vascular smooth muscle cells [8].

GH replacement therapy in patients previously treated for CNS tumors is subject not only to oncological assessments, but also to auxological and endocrinological parameters (growth failure, IGF-1 levels, pituitary deficiencies, skeletal growth), and to the results of arginine and/or clonidine challenges.

## Case presentation

We report the case of a 7-year-old girl with metastatic medulloblastoma (T3b M1 according to the Chang staging system) diagnosed as a result of an epileptic seizure after 3 months of headache. Brain MRI in July 2000 revealed a mass in the posterior fossa, in the midline cerebellum next to the fourth ventricle and vermis, causing hydrocephalus and an initial herniation of the cerebellar tonsils. At the end of July, she underwent a macroscopically complete resection of a 3 × 3 cm nodule filling the fourth ventricle and attached to its lateral recesses. The final histologic diagnosis was classic medulloblastoma.

A postoperative cerebral and spinal MRI in August 2000 revealed an ambiguous neoplastic residue close to the surgical cavity, jutting out into the fourth ventricle, and malignant neoplastic cells were found in the cerebrospinal fluid.

From August 2000 to March 2001, the girl was treated according to our institutional protocol for metastatic medulloblastoma/PNET in children over 3 years of age, which included sequential high-dose chemotherapy, radiotherapy, and myeloblative-dose chemotherapy with autologous hemopoietic stem cell rescue [9]. The protocol was partially modified omitting two scheduled vincristine administrations due to intestinal toxicity after the first course.

Hyperfractionated accelerated radiotherapy was administered from November to December 2000, scheduled in two daily 1.3 Gy fractions 6 hours apart, 5 days a week for a total dose of 31.2 Gy to the craniospinal axis plus a boost to the posterior fossa using a 1.5 Gy fractionation twice a day to give a total dose of 59.7 Gy.

Diagnostic lumbar puncture before radiotherapy still documented malignant neoplastic cells, while lumbar puncture and MRI after radiotherapy and between the two myeloablative courses were both negative, with the latter showing only surgical sequelae.

In June 2001, our patient started the follow-up program with routine MRI and outpatient visits. In September 2001, MRI showed multiple focal contrast-enhanced areas in the right thalamus, brainstem, and cerebellum, interpreted as iatrogenic alterations. A spectrum MRI confirmed their necrotic and treatment-related nature. These lesions had increased in number in the MRI of December 2001, and they appeared more strongly gadolinium-enhanced in subsequent images in February and April 2002. Cortisone therapy was administered from January 2002, starting with 6 mg/day. It was then progressively tapered off, but the neurological signs became worse, with cervical pain and frontal headache, so it was re-introduced from March to July 2002.

In March 2002, a year after ending the oncological treatment, an endocrinologist assessed the girl. At 8 years and 4 months old, her growth characteristics were: height 119 cm (3^rd ^percentile), growth target 160 cm (25^th ^percentile), growth rate 0 cm per year, and basal IGF-1 below the 3^rd ^percentile. Since her growth had not recovered, GH replacement therapy was started after an arginine challenge in February 2004, administering somatotropin at a dosage of 5 mg per week (15 UI, 0.16 mg/Kg per week).

In May 2004 (3 months after starting somatotropin), MRI showed a clear progression of the enhanced lesions distributed along the occipital horn of the right lateral ventricle and the peritrigonal region. The main finding was perifocal edema, already seen at previous follow-up scans but now extending further, to the posterior half of the right hemisphere and in contact with the basal nuclei and the thalamus. There was no evidence of recurrent disease in the posterior fossa. The girl also reported headache and visual impairment.

**Figure 1:**
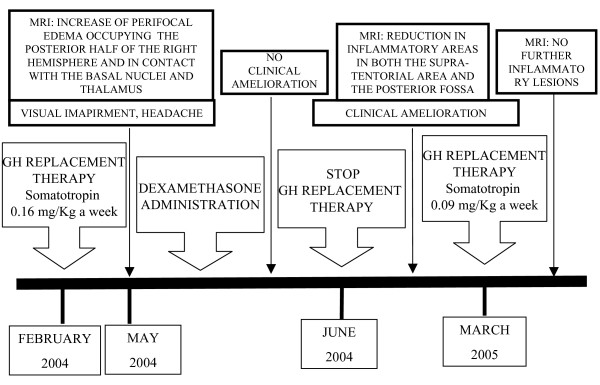
**Relationship between somatotropin administration, MRI, clinical outcome, and steroid administration**. Growth hormone administration was related to both MRI changes and a worse clinical performance. Dexamethasone administration produced no amelioration, either clinical or visible in the MRI. This was achieved instead by first suspending somatotropin, then restoring it at lower doses.

**Figure 2:**
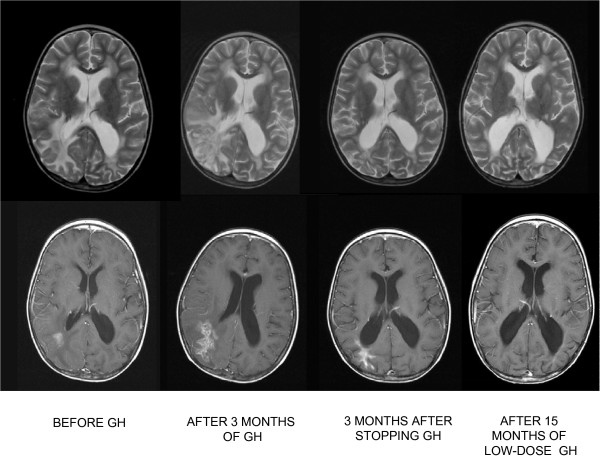
**Iconography strongly suggests an association between growth hormone administration and proinflammatory activity**. Axial MRI images immediately before administering growth hormone, after 3 months of growth hormone administration, 3 months after suspending growth hormone, and after 15 months of lower-dose administration of growth hormone. The areas of focal enhancement in the images taken before the therapy with growth hormone reveal inflammation and necrosis, an expression of post-therapy alterations. After 3 months of growth hormone therapy, the main finding is an increase in the perifocal edema occupying the posterior half of the right hemisphere, which decreased after suspending growth hormone. After 15 months of low-dose administration of growth hormone, there is no evidence of further edema in the post-therapy radiological alterations.

Cortisone therapy was administered again, with no improvement in the visual impairment or MRI findings. Due to the latter, and the possible proinflammatory activity of GH, somatotropin was discontinued in June 2004. Subsequent MRIs in September 2004 (3 months after suspending somatotropin) and January 2005 (after 7 months without somatotropin) showed a remarkable reduction of the inflammatory areas both in the supratentorial region and in the posterior fossa; the visual impairment had also improved after suspending GH. In March 2005, we introduced somatotropin again at lower doses (3 mg a week) with no evidence of further edema in the post-therapy alterations at subsequent MRIs in November 2005, June 2006, December 2006, July 2007, and March 2008 (Figures 1 and 2). No recurrent disease was documented by these MRI studies, and the girl is currently in continuous complete response.

## Conclusion

GH replacement therapy has proved effective in ameliorating growth impairment and quality of life, as evaluated by both health- and psychological well-being tools. If GH replacement is continued even after the final statural growth has been achieved, it also seems to have positive effects on somatic and skeletal maturation [2,3].

The experience we describe in this case report is significant because many reports document the safety of GH replacement therapy in terms of tumor progression, recurrence, or development of secondary neoplasms, but few have documented MRI changes and related symptoms due to an exacerbation of any inflammatory parenchymal lesions while on GH replacement therapy.

Moreover, the improved tools available for treating CNS tumors (including radiotherapy and myeloablative chemotherapy with autologous stem cell transplantation) have made it more difficult to obtain a differential diagnosis on MRI findings because it is not clear how these therapeutic measures and their late effects might affect the radiological process [10]. The case reported here, and the related iconography, are strongly suggestive of a close association between GH administration and proinflammatory activity; although more evidence is needed to support our observations.

This case also suggests that GH replacement therapy should be delivered with caution and should be closely monitored in patients previously treated for CNS malignancies.

## Abbreviations

GH: growth hormone; GHD: growth-hormone deficit; CNS: central nervous system; ACTH: adrenocorticotropic hormone; TSH: thyroid-stimulating hormone.

## Consent

Written informed consent was obtained from the patient's parents for publication of this case report and any accompanying images. A copy of the written consent is available for review by the Editor-in-Chief of this journal.

## Competing interests

The authors declare that they have no competing interests.

## Authors' contributions

VB and MM are the pediatric oncologists who had the patient in their care during the oncological treatment and follow-up; they drafted the main part of the manuscript. FS is the pediatric oncologist who reviewed all the MRIs to choose the most representative and who wrote the figure legends.

LG is the radiotherapist who treated the girl and provided important information on radiotherapy-induced sequelae. FP and AM are the endocrinologists responsible for the arginine challenge to identify the girl's GH deficiency. ES is the endocrinologist who collected the results of the arginine challenges and designed the replacement therapy. EB provided us with current knowledge on GH proinflammatory activity.
